# Phenotyping Adherence Through Technology-Enabled Reports and Navigation (the PATTERN Study): Qualitative Study for Intervention Adaptation Using the Exploration, Preparation, Implementation, and Sustainment Framework

**DOI:** 10.2196/54916

**Published:** 2024-10-17

**Authors:** Allison P Pack, Stacy C Bailey, Rachel O'Conor, Evelyn Velazquez, Guisselle Wismer, Fangyu Yeh, Laura M Curtis, Kenya Alcantara, Michael S Wolf

**Affiliations:** 1 Division of General Internal Medicine Feinberg School of Medicine Northwestern University Chicago, IL United States

**Keywords:** older adults, polypharmacy, medication adherence, intervention development, qualitative research

## Abstract

**Background:**

Older adults with multiple chronic conditions (MCC) and polypharmacy often face challenges with medication adherence. Nonadherence can lead to suboptimal treatment outcomes, adverse drug events, and poor quality of life.

**Objective:**

To facilitate medication adherence among older adults with MCC and polypharmacy in primary care, we are adapting a technology-enabled intervention previously implemented in a specialty clinic. The objective of this study was to obtain multilevel feedback to inform the adaptation of the proposed intervention (Phenotyping Adherence Through Technology-Enabled Reports and Navigation [PATTERN]).

**Methods:**

We conducted a formative qualitative study among patients, clinicians, and clinic administrators affiliated with a large academic health center in Chicago, Illinois. Patient eligibility included being aged 65 years or older, living with MCC, and contending with polypharmacy. Eligibility criteria for clinicians and administrators included being employed by any primary care clinic affiliated with the participating health center. Individual semistructured interviews were conducted remotely by a trained member of the study team using interview guides informed by the Exploration, Preparation, Implementation, and Sustainment Framework. Thematic analysis of interview audio recordings drew from the Rapid Identification of Themes from Audio Recordings procedures.

**Results:**

In total, we conducted 25 interviews, including 12 with clinicians and administrators, and 13 with patients. Thematic analysis revealed participants largely found the idea of technology-based medication adherence monitoring to be acceptable and appropriate for the target population in primary care, although several concerns were raised; we discuss these in detail.

**Conclusions:**

Our medication adherence monitoring intervention, adapted from specialty care, will be implemented in primary care. Formative interviews, informed by the Exploration, Preparation, Implementation, and Sustainment Framework and conducted among patients, clinicians, and administrators, have identified intervention adaptation needs. Results from this study could inform other interventions using the patient portal with older adults.

## Introduction

Older adults are more likely to have multiple chronic conditions (MCC) and associated polypharmacy [1,2]. As a result, problems with treatment adherence are common; previous estimates have found 30% to 50% of community-dwelling older adults aged 65 years or older demonstrate poor medication adherence [3-5]. These individuals are at subsequent greater risk for suboptimal treatment benefits and adverse drug events [6-8]. Studies have also shown that inadequate adherence is associated with poorer health-related quality of life [9-11], higher health care costs [10,12], and increased mortality risk [13,14].

The model of medication self-management has facilitated the deconstruction of patient medication use and was further used as a model to categorize adherence barriers as cognitive (eg, forgetfulness), psychological (eg, health literacy, depression, and motivation), medical (eg, acute illness), regimen (eg, side effects and complex dosing schedules), social (eg, transportation and support), and economic (eg, cost) [15]. Given this heterogeneity of challenges, interventions designed to address them should be informed by a patient’s own report of perceived barriers.

To this end, we are adapting the “Transplant regimen Adherence in Kidney recipients by Engaging Information Technologies” (TAKE-IT) strategy [16,17]. TAKE-IT was a successful program designed for routinely monitoring medication use and mobilizing resources for medication adherence support in the transplant center among kidney transplant recipients. More specifically, kidney transplant recipients were sent monthly brief medication adherence assessments to complete through a secure portal linked to the electronic medical record. Results from the assessments identified if recipients were experiencing a medication adherence challenge, and if so, which type. This information was automatically sent to the transplant center as a notification within the electronic health record; staff addressed concerns as appropriate with existing center resources.

Using TAKE-IT as a model, our adapted intervention, renamed as PATTERN (Phenotyping Adherence Through Technology-Enabled Reports and Navigation), will be pilot-tested for acceptability, appropriateness, and feasibility in primary care. The initial conception of PATTERN was to target patients aged 65 years or older who are living with MCC and polypharmacy (using Medicare Part D medication therapy management criteria of ≥8 medications). We chose this target population based on their propensity to experience medication adherence challenges within primary care. However, for all other adaptations, we chose to rely on information gathered as part of this formative, qualitative study.

This study presents findings from our interviews with clinicians, administrators, and patients. Interview objectives were to explore intervention acceptability and appropriateness and gather suggestions for adaptation and eventual pilot implementation.

## Methods

### Study Design

We conducted a formative, qualitative study in 2023.

### Eligibility Criteria, Recruitment, and Setting

Clinicians and clinic administrators were eligible to participate if they were currently employed as a primary care clinician or administrator at any primary care clinic affiliated with the participating academic medical center in Chicago, Illinois. These individuals (herein referred to as clinicians) were identified using existing internal networks and sent an email by members of the research team informing them of the study. Those interested in participating subsequently contacted the study staff and provided informed consent. Interviews were scheduled at their convenience.

Primary care patients from the same academic medical center as clinicians were eligible to participate if they were aged 65 years or older; had a chart diagnosis of diabetes, hypertension, and hyperlipidemia; and were prescribed 8 or more medications. Given their experiences with MCC and polypharmacy, patients meeting these criteria reflected our target population for the PATTERN intervention study. Potentially eligible patient participants were mailed a letter about the study and offered an opportunity to opt out of further contact from the research team. Those who did not opt out were phoned by a research coordinator who provided additional study details. Individuals who expressed interest in participating were screened for eligibility criteria, engaged in the informed consent process, and interviewed at their convenience.

### Theoretical Framework and Data Collection

Before data collection, interviewers informed participants about the initial PATTERN intervention idea and offered them an opportunity to ask clarifying questions (Multimedia Appendices 1 and 2). Next, interviewers trained in qualitative data collection conducted audio-recorded interviews remotely over secure videoconferencing software; to facilitate this, they who used in-depth interview guides informed by the Exploration, Preparation, Implementation, and Sustainment (EPIS) Framework [18]. Separate guides were created for clinicians and patient participants, although both guides included questions pertaining to the exploration and early planning phases of the EPIS Framework (Multimedia Appendices 1 and 2). EPIS is an implementation science framework designed to facilitate multiple phases of intervention implementation. For the exploration and early planning phases, the framework helps to guide adoption and adaptation decisions by considering the needs and opinions of multiple key stakeholders, including clinicians and patients. In general, questions broached (1) the appropriateness of clinical indications for the target population, (2) the extent to which the planned intervention fits the needs of the target population, and (3) specific features of the intervention; for clinicians, questions also broached (4) competing job demands and (5) the perceived readiness for affiliated primary care clinics to implement the intervention. All participants also completed brief demographic questionnaires with data captured in REDCap (Research Electronic Data Capture; Vanderbilt University) software. At the conclusion of each interview, the interviewers wrote detailed memos [19].

### Analysis

We used descriptive statistics to examine the demographic data of our samples. To conduct our qualitative analyses, we drew from the Rapid Identification of Themes from Audio Recordings procedures [20]. This entailed creating an Excel (Microsoft) matrix with discrete rows representing individual participants. The columns represented a priori codes identified from our in-depth interview guide and emergent codes from our review of postinterview memos [19]. For the first 6 interviews (3 clinicians and 3 patients), 2 coders listened to the audio recordings and completed the matrix; this involved summarizing what separate participants said related to each code and transcribing illustrative quotes verbatim. Coders then reviewed this “double coding” and resolved discrepancies to ensure information was accurately captured for each participant and that all codes were consistently applied. Coders individually coded the remaining interviews. Once coding was complete, coders summarized the data across participants to reveal relevant and code-specific themes [19,21].

### Ethical Considerations

The Northwestern University’s Institutional Review Board (IRB) approved all study procedures (STU00217555). Participants provided informed consent and data were de-identified. Patients were compensated US $30, and clinicians were compensated US $100 for their participation in this study.

## Results

### Participant Characteristics

Participant characteristics are presented in Tables 1 and 2. A total of 12 clinicians and 13 patient participants were enrolled; these sample sizes are considered sufficient for reaching thematic saturation [22]. Clinician participants largely included physicians or nurse practitioners (11/12, 92%). Most were female (9/12, 75%) and identified as White (10/12, 84%). A majority also reported practicing in a downtown location (8/12, 71%), although years of practice ranged between fewer than 5 years and 20 or more years. Patient participants ranged in age from 65 years to 84 years. Just over half (7/13, 54%) of the patients were female and most identified as White (10/13, 77%); marital status and self-reported income varied widely.

**Table 1:** Characteristics of clinicians (n=12) who participated in formative in-depth interviews between February and June of 2023.

Demographics		Value, n (%)
**Job title**		
	Physician or nurse practitioner	11 (92)
	Clinic administrator	1 (8)
**Practice (years)**		
	≤5	2 (18)
	6-10	2 (18)
	11-15	1 (9)
	16-20	2 (18)
	≥20	4 (36)
**Clinic location**		
	Downtown	8 (71)
	Suburban area	3 (27)
	No specific site	1 (9)
**Sex**		
	Male	3 (25)
	Female	9 (75)
**Ethnicity**		
	Hispanic	0 (0)
	Non-Hispanic	12 (100)
**Race**		
	White	10 (83)
	Asian	1 (8)
	Prefer not to answer	1 (8)

**Table 2:** Characteristics of patients (n=13) who participated in formative in-depth interviews in June of 2023.

Characteristics			Value
**Sex, n (%)**			
	Male	6 (46)	
	Female	7 (54)	
**Age (years)**			
	Mean (SD)	70.9 (6)	
	Median (Range)	68 (65-84)	
**Ethnicity, n (%)**			
	Hispanic	0 (0)	
	Non-Hispanic	13 (100)	
**Race, n (%)**			
	White	10 (77)	
	Black or African American	2 (15)	
	Other	1 (8)	
**Marital status, n (%)**			
	Married	4 (31)	
	Widowed	3 (23)	
	Divorced	3 (23)	
	Single	3 (23)	
**Income (US $), n (%)**			
	≤25,000	1 (8)	
	25,000-49,999	5 (38)	
	50,000-99,999	4 (31)	
	100,000-199,000	1 (8)	
	≥200,000	2 (15)	

### Thematic Findings

Results from our thematic analysis are presented below under the following key domains: (1) acceptability and appropriateness of the PATTERN intervention, (2) perceived concerns about the intervention, (3) current practices and clinician awareness of medication adherence challenges, and finally, (4) clinic readiness and considerations for readiness.

#### Acceptability and Appropriateness of the PATTERN Intervention

##### Most Participants Reported Acceptability of Technology-Based Medication Adherence Monitoring

All clinicians thought the idea of identifying and categorizing adherence concerns would be useful for patient care, particularly for patients with diabetes, hypertension, and hyperlipidemia. One clinician explained:

I think it would definitely benefit our awareness of adherence issues.

Female clinician, 16 to 19 years of experience

And another noted how the intervention was appropriate for clinical practice:

I see it fitting into my practice nicely...I wish we did this...It’s super important

Female clinician, 5 or fewer years of experience

Similarly, most patient participants revealed they thought older adults with similar conditions to theirs, and those who struggle with medication adherence, would benefit from intervention:

Any patient that takes multiple medications would benefit from something [like this].

67-year-old woman

When asked whether they themselves might benefit from the medication adherence intervention, over half of all patient participants indicated they would. Some explained they personally struggle with forgetfulness, confusion, or a lack of motivation, while others noted they could use additional support organizing or following their medication regimens. For example, 1 participant described the challenge of managing multiple medications, not only for himself, but also for his wife:

I manage mine and my wife’s [medications]. So, it gets to be...confusing at times...

82-year-old man

This added complexity made it challenging for him to ensure he was taking his own medications as prescribed.

#### Perceived Concerns About the Intervention

##### Patient Portal Usage Is High Among the Target Population but Could Be a Challenge for Some

Some clinicians revealed patients in the target population can and do use the patient portal. One clinician explained how portal usage is increasingly common and useful for gathering patient reported outcomes data before annual wellness visits:

I don’t think there will be a barrier there - we actually have pretty high involvement of our 65 and up population on MyChart. I think we have to make sure it’s simple. But if it’s in the questionnaire format that are used to getting, I think we’re getting decent return on those from our patients. We’re doing that for our annual wellness visits.”

Male clinician, 20 or more years of experience

However, clinicians also noted that it will be important to verify who is completing the adherence assessments, as many older adults who use the portal have a “proxy user” who manages their account on their behalf. As 1 clinician explained:

I think that’s going to be tricky - figuring out who’s actually filling it out...it’s not always clear. You’re going to have to identify who’s doing it.

Female clinician, 20 or more years of experience

When asked what challenges patient participants believed “others” may have in completing adherence assessments through the patient portal, they often cited challenges in technology access or use:

...For somebody that doesn’t like to use a computer, or [a smart phone]...It would be more of a challenge...

77-year-old woman

Similarly, some patients noted others may not have access to an appropriate device (ie, computer, tablet, and smart phone) or know how to navigate the portal and the adherence assessment.

##### Monthly Delivery of Adherence Assessments May Not Be Necessary for Primary Care Patients

Nearly half of the clinicians perceived the monthly delivery of adherence assessments may not be necessary or appropriate for the target population in primary care. They suggested tying assessments to upcoming visits or an annual wellness visit or delivering it every 3 months; this way, they explained, identified adherence concerns are more likely to be addressed efficiently:

If it were done prior to an office visit, it might fit better into workflow than if it’s ongoing outside...If you tell me your patient has these challenges taking medications and you’re going to see them in 3 days, then I can incorporate that into what I’m doing with them during that office visit. But if you’ve just sent me a message about this and their appointment is in 4 months, that’s going to be less well received...Even if I have to go back and forth with another team member asynchronously for a large number of patients, I’m starting to feel overwhelmed.

Male clinician, 20 or more years of experience

Among patient participants, however, all noted that they would be willing to complete monthly adherence assessments through the patient portal. Nearly all these patients explained they are comfortable accessing and using their accounts. Some suggested limited reminders by text or the portal may be useful to help them complete the assessments. Nevertheless, 1 participant noted she may need specific assistance accessing her portal account as she is an infrequent user.

##### Notifications Have the Potential to Overwhelm Physicians

Several clinicians noted physicians are already overwhelmed with current workloads. They worried physicians may feel additional burden if the intervention results in the receipt of a large volume of notifications or messages to the clinic through the patient portal.

The challenges are...we get a lot of inbox messages...sometimes you get inbox fatigue.

Female clinician, 5 or fewer years of experience

This participant, and others, suggested it would be important to minimize notifications specifically targeting physicians.

##### Word Choice on the Adherence Assessments Has the Potential to Be Off-Putting

A couple of clinicians noted the importance of centering the patient and being mindful of how they will perceive the language used. These clinicians worried language about adherence challenges could be off-putting to older patients who are concerned about maintaining independence. For example, one explained:

I think it’s going to be the wording too. Because you can’t just say, “Hey do you need help?” because nobody likes that idea. Or you can’t say, “Hey do you find this challenging?” because that implies that you’re having a problem with your independence.

Female clinician, 20 or more years of experience

This concern was echoed by a few patient participants who perceived long, or insensitive assessments could be a deterrent to portal completion among older adults.

#### Current Practices and Clinician Awareness of Medication Adherence Challenges

##### Perceptions Differed on Whether Clinicians Know When Patients Have Adherence Challenges

Most clinicians perceived clinicians do not always know when patients are experiencing adherence challenges, with a couple explaining that patients sometimes hide adherence challenges. For example, 1 clinician noted:

I think that’s the first step [awareness]. It’s that we don’t even necessarily know that they’re having these issues because either they don’t tell us, they’re embarrassed, or they don’t realize they’re doing it wrong...

Female clinician, 16 to 19 years of experience

Others indicated it can be difficult to address all competing priorities within the span of a single primary care visit, as such, there are times when some concerns are not discussed.

The smaller number of clinicians who indicated clinicians are typically aware, explained that home visits, lab tests, and taking the time to ask the appropriate questions can each provide useful insight into potential adherence challenges. Most often, however, they noted that unfilled prescriptions are a key indicator of potential adherence challenges.

Patient participants, on the other hand, largely perceived their health care teams are aware of medication adherence challenges. These patients explained that they discuss adherence concerns with their health care teams and strategize solutions, particularly when it pertains to accessing or changing prescription medications. For example, 1 patient noted:

They do [know when I have adherence challenges]...we have conversations about it, and we work together.

84-year-old White woman

Patients also suggested that results from laboratory tests can prompt discussions or actions to address adherence concerns.

##### Perceptions Differed on Whether Clinicians Know the Type of Challenge Patients Have

When asked whether clinicians typically know the type of medication adherence challenge a patient is experiencing, most clinicians perceived clinicians do not. Explanations included that clinicians do not always have enough time to ask the necessary questions during a busy visit, and that patients themselves do not always know; as such, they do not report it to their care teams.

Nevertheless, some clinicians revealed that it may be easier to identify some types of adherence challenges than others. Cognitive impairment was considered more “obvious” to identify, while economic challenges were most often noted as ones that can “slip through the cracks”. One clinician explained:

Patients...they’re people. They want others to believe that they’re fully capable. And sometimes the admission that they just don’t have the resources, the money, to do things can be a challenge. Others are quite honest about it “I can’t take this medication; it’s too expensive.”

Male clinician, 20 or more years of experience

Conversely, most patient participants perceived their health care teams do know the type of medication adherence challenges they experience, not necessarily because of something the health care team is doing, but because the patients themselves are reporting their challenges. One patient explained she felt comfortable initiating these discussions with her health care team because of the relationship she has with them:

I think they’re well informed, yes...There’s a good relationship there.

74-year-old woman

Several patients were unsure whether their health care teams were aware of the type of challenges they experienced. One man explained his uncertainty to a lack of discussion with his health care team:

I mean they just will prescribe it and just assume I’m taking it...I really don’t know

70-year-old man

A couple of others recognized, similar to clinicians, that there is not always sufficient time to discuss challenges in a clinic visit, and that it can be difficult to disclose economic challenges. For example, one man noted:

I honestly don’t think they have time to worry about each individual...other than to ask a question and hurry on to the next one

82-year-old man

#### Clinic Readiness and Considerations for Enhancing Readiness

##### Clinic Readiness for This Intervention may Depend on Staffing and the Number of Patients Who Will Receive the Intervention

Clinicians had mixed feelings about how ready their clinics might be to implement an intervention like PATTERN. A couple perceived their clinics were ready because, to some extent, they are doing similar work collecting patient reported outcomes related to social determinants of health; moreover, they are relying on social workers to address specific identified challenges.

Nevertheless, some clinicians felt strongly they were not yet ready for an intervention like PATTERN because staffing would be a critical challenge if many patients are to receive the intervention. One clinician explained:

We don’t have the social worker manpower for this...maybe the hospital does, but that would have to be addressed. And I don’t know if we have the pharmacist manpower. It depends on how many patients.

Female clinician, 11 to 14 years of experience

##### To Reduce the Potential for Overwhelming Physicians, Ensure Adherence Assessment Results Are Tied to Clear Guidance

When adherence assessment results are sent to the clinic, some clinicians suggested that there needs to be clear guidance on how the clinic should respond; otherwise, it is likely to create additional burden for physicians who are already over worked and feeling “overwhelmed”. If possible, these participants felt that results could largely be fielded by someone else (potentially a triage nurse or, ideally, a clinical pharmacist if available); this person could then direct appropriate others to respond. One clinician explained that guidance is necessary to ensure timely action is taken:

This would be good for my patients – and if it’s good for my patients, it’s good for me. My fear is...telling docs that they’re missing something – like, that’s how we might see it. “Oh, this person has a cognitive issue and they’re not able to take their meds. Just wanted you to know!” That feels like one more thing that’s my responsibility.“

Female clinician, 16 to 19 years of experience

## Discussion

### Principal Findings

We conducted a formative qualitative study with clinicians and patients to explore the acceptability and appropriateness of the PATTERN intervention, and to gather suggestions to guide adaptation and future implementation.

Participants in this study largely perceived the target population could benefit from technology-based medication adherence monitoring. Other studies conducted among older adults have found similar findings, suggesting technology-based adherence monitoring may be a useful approach for this population [23]. Interestingly, however, there was discrepancy among clinicians and patients in our sample regarding whether clinicians could benefit from reports detailing the categorized adherence concerns of patients. Clinicians perceived these reports would be useful, while patients perceived their clinicians are already aware of their medication adherence challenges. Nevertheless, some studies have found patients hold more favorable views of their clinicians than their clinicians hold of themselves [24]. Ensuring clinicians and patients both have accurate assessments of medication adherence challenges is critical to improving care and health outcomes [25].

Furthermore, there was a perception that the target population is capable of using and accessing the patient portal. Some concerns were raised as to whether all individuals will have the capacity to access and use the patient portal; however, patient portals are becoming ubiquitous [26]. In a systematic review, patient portal interventions were shown to be effective at enhancing medication adherence among a variety of populations [27]. Therefore, to reduce the potential for exacerbating existing health inequities, numerous studies have been conducted to examine barriers and facilitators of portal use among older adults, with many suggesting that encouragement from clinicians may be a substantial facilitator [28,29]. Nevertheless, efforts may be needed to identify whether the intended individual or a proxy user is completing the portal questionnaire. Other studies are currently examining how best to identify and engage proxy users [30].

Participants in this study also offered several suggestions to enhance the uptake and maintained use of the intervention. First, participants suggested the need for clear instructions to guide patients on how to access and complete the adherence assessments. A similar study conducted using focus groups with patients and clinicians also indicated patient-friendly training materials may help users better navigate health information provided through patient portals [31]. Furthermore, health literacy best practices have been applied in many other studies to ensure patients can read, understand, and complete the assessments, as well as to ensure the language is polite and respectful [32-36]. Other research also suggests tailoring portal information to individual care needs may result in increased portal use among older adults [37]. Second, although patient participants indicated they would be open to receiving and completing monthly adherence assessments, clinicians suggested that this cadence may be unnecessary and potentially burdensome for clinical care teams to accommodate. Instead, the recommendation provided was to tie assessments to upcoming primary care visits. This change would enable clinical care teams to more efficiently address medication adherence concerns and would likely reduce the number of notifications sent to the clinic. Previsit and same-day completion of patient reported outcomes are feasible and useful for patients with a variety of health conditions [37-39].

Finally, clinicians similarly suggested the need to ensure assessment results are actionable and that resources can be mobilized to address them. They explained that many clinics are currently under-resourced, which could potentially challenge implementation uptake and effectiveness. However, if clear guidance were provided on how to address specific adherence concerns, clinics may be more willing to implement the intervention. For example, if a triage nurse is identified to field all identified adherence concerns, they should receive clear instructions on what to do, or who to contact, for each type of concern, whether that be cognitive, psychological, medical, regimen-related, social, or economic. This was likewise noted by a study that aimed to examine whether sharing patient reported outcomes with clinicians improves patient symptoms. Results indicated that providing information alone was insufficient; as such, the authors suggested clinician training may be needed [40]. Training was also requested by clinician participants in another qualitative study; they reported standardized protocols are needed to help them better manage and respond to patient needs through the portal [31].

### Limitations

This study is not without limitations. We conducted a formative and cross-sectional qualitative study among clinicians and patients from a large academic health center in Chicago; results are therefore not generalizable. Furthermore, our sample lacks racial and ethnic diversity; that said, it is largely reflective of the target population at the participating academic health center where the intervention will eventually be delivered. Additional adaptation may be needed for implementation elsewhere.

### Conclusion

In conclusion, while the PATTERN intervention was perceived by clinicians and patient participants as being acceptable and appropriate, specific recommendations, guided by the EPIS framework, were provided for intervention adaptation and eventual implementation. Adaptation is currently underway, and the optimized intervention will subsequently be pilot-tested in primary care. Lessons learned from this formative study could inform other interventions designed to assess patient reported outcomes through the patient portal.

## Appendix

Multimedia Appendix 1 COREQ (Consolidated Criteria for Reporting Qualitative Research) checklist.

Multimedia Appendix 2 Background and interview guides.

## Abbreviations

EPIS: Exploration, Preparation, Implementation, and Sustainment
MCC: multiple chronic conditions
NIH: National Institutes of Health
PATTERN: Phenotyping Adherence Through Technology-Enabled Reports and Navigation
REDCap: Research Electronic Data Capture
TAKE-IT: Transplant regimen Adherence in Kidney recipients by Engaging Information Technologies

## Data Availability

The data sets generated during or analyzed during this study are not publicly available, as the audio files could potentially identify our participants; deidentified transcripts were not generated.

## References

[1] Bishop NJ, Haas SA, Quiñones Ana R (2022). Cohort trends in the burden of multiple chronic conditions among aging U.S. adults. J Gerontol B Psychol Sci Soc Sci.

[2] Delara M, Murray L, Jafari B, Bahji A, Goodarzi Z, Kirkham J, Chowdhury M, Seitz DP (2022). Prevalence and factors associated with polypharmacy: a systematic review and Meta-analysis. BMC Geriatr.

[3] Conn VS, Ruppar TM, Chan KC, Dunbar-Jacob J, Pepper GA, de Geest S (2015). Packaging interventions to increase medication adherence: systematic review and meta-analysis. Curr Med Res Opin.

[4] Maher RL, Hanlon J, Hajjar ER (2014). Clinical consequences of polypharmacy in elderly. Expert Opin Drug Saf.

[5] Marcum ZA, Sevick MA, Handler SM (2013). Medication nonadherence: a diagnosable and treatable medical condition. JAMA.

[6] Sarkar U, López Andrea, Maselli JH, Gonzales R (2011). Adverse drug events in U.S. adult ambulatory medical care. Health Serv Res.

[7] Gurwitz JH, Field TS, Avorn J, McCormick D, Jain S, Eckler M, Benser M, Edmondson AC, Bates DW (2000). Incidence and preventability of adverse drug events in nursing homes. Am J Med.

[8] Zhang M, Holman CDJ, Preen DB, Brameld K (2007). Repeat adverse drug reactions causing hospitalization in older Australians: a population-based longitudinal study 1980-2003. Br J Clin Pharmacol.

[9] Makovski TT, Schmitz S, Zeegers MP, Stranges S, van den Akker M (2019). Multimorbidity and quality of life: systematic literature review and meta-analysis. Ageing Res Rev.

[10] Marengoni A, Angleman S, Melis R, Mangialasche F, Karp A, Garmen A, Meinow B, Fratiglioni L (2011). Aging with multimorbidity: a systematic review of the literature. Ageing Res Rev.

[11] Gray SL, Marcum ZA, Schmader KE, Hanlon JT (2018). Update on medication use quality and safety in older adults, 2017. J Am Geriatr Soc.

[12] Cutler RL, Fernandez-Llimos F, Frommer M, Benrimoj C, Garcia-Cardenas V (2018). Economic impact of medication non-adherence by disease groups: a systematic review. BMJ Open.

[13] Nunes BP, Flores TR, Mielke GI, Thumé Elaine, Facchini LA (2016). Multimorbidity and mortality in older adults: a systematic review and meta-analysis. Arch Gerontol Geriatr.

[14] Walsh CA, Cahir C, Tecklenborg S, Byrne C, Culbertson MA, Bennett KE (2019). The association between medication non-adherence and adverse health outcomes in ageing populations: a systematic review and meta-analysis. Br J Clin Pharmacol.

[15] Bailey SC, Oramasionwu CU, Wolf MS (2013). Rethinking adherence: a health literacy-informed model of medication self-management. J Health Commun.

[16] Serper M, Ladner DP, Curtis LM, Nair SS, Hur SI, Kwasny MJ, Ho B, Friedewald J, Reese PP, Abecassis MMI, Wolf MS (2021). Transplant regimen adherence for kidney recipients by engaging information technologies (TAKE IT): rationale and methods for a randomized controlled trial of a strategy to promote medication adherence among transplant recipients. Contemp Clin Trials.

[17] Yoon ES, Hur S, Curtis LM, Wynia AH, Zheng P, Nair SS, Bailey SC, Serper M, Reese PP, Ladner DP, Wolf MS (2022). A multifaceted intervention to improve medication adherence in kidney transplant recipients: an exploratory analysis of the fidelity of the TAKE IT trial. JMIR Form Res.

[18] Aarons GA, Hurlburt M, Horwitz SM (2011). Advancing a conceptual model of evidence-based practice implementation in public service sectors. Adm Policy Ment Health.

[19] Ulin PR, Tolley EE, Mack N, Robinson ET, Succop SM (2005). Qualitative Methods in Public Health: A Field Guide for Applied Research, 2nd Edition.

[20] Neal JW, Neal ZP, VanDyke E, Kornbluh M (2014). Expediting the analysis of qualitative data in evaluation. Am J Eval.

[21] Gale NK, Heath G, Cameron E, Rashid S, Redwood S (2013). Using the framework method for the analysis of qualitative data in multi-disciplinary health research. BMC Med Res Methodol.

[22] Hennink M, Kaiser BN (2022). Sample sizes for saturation in qualitative research: a systematic review of empirical tests. Soc Sci Med.

[23] Park LG, Ng F, K Shim J, Elnaggar A, Villero O (2020). Perceptions and experiences of using mobile technology for medication adherence among older adults with coronary heart disease: a qualitative study. Digit Health.

[24] Hoffstädt Hinke, Stouthard J, Meijers MC, Westendorp J, Henselmans I, Spreeuwenberg P, de Jong P, van Dulmen S, van Vliet LM (2020). Patients' and clinicians' perceptions of clinician-expressed empathy in advanced cancer consultations and associations with patient outcomes. Palliat Med Rep.

[25] Fürthauer Johanna, Flamm M, Sönnichsen Andreas (2013). Patient and physician related factors of adherence to evidence based guidelines in diabetes mellitus type 2, cardiovascular disease and prevention: a cross sectional study. BMC Fam Pract.

[26] Carini E, Villani L, Pezzullo AM, Gentili A, Barbara A, Ricciardi W, Boccia S (2021). The Impact of Digital Patient Portals on Health Outcomes, System Efficiency, and Patient Attitudes: Updated Systematic Literature Review. J Med Internet Res.

[27] Han H, Gleason KT, Sun C, Miller HN, Kang SJ, Chow S, Anderson R, Nagy P, Bauer T (2019). Using patient portals to improve patient outcomes: systematic review. JMIR Hum Factors.

[28] Sakaguchi-Tang DK, Bosold AL, Choi YK, Turner AM (2017). Patient portal use and experience among older adults: systematic review. JMIR Med Inform.

[29] Yoon E, Hur S, Opsasnick L, Huang W, Batio S, Curtis LM, Benavente JY, Lewis-Thames MW, Liebovitz DM, Wolf MS, Serper M (2024). Disparities in patient portal use among adults with chronic conditions. JAMA Netw Open.

[30] Dukhanin V, Wolff JL, Salmi L, Harcourt K, Wachenheim D, Byock I, Gonzales MJ, Niehus D, Parshley M, Reay C, Epstein S, Mohile S, Farrell TW, Supiano MA, Jajodia A, DesRoches CM (2023). Co-designing an initiative to increase shared access to older adults' patient portals: stakeholder engagement. J Med Internet Res.

[31] Hefner JL, Sieck CJ, Walker DM (2022). Patient and physician perspectives on training to improve communication through secure messaging: clarifying the rules of engagement. Health Care Manage Rev.

[32] Shoemaker SJ, Wolf MS, Brach C (2014). Development of the patient education materials assessment tool (PEMAT): a new measure of understandability and actionability for print and audiovisual patient information. Patient Educ Couns.

[33] Reading Turchioe M, Mangal S (2024). Health literacy, numeracy, graph literacy, and digital literacy: an overview of definitions, evaluation methods, and best practices. Eur J Cardiovasc Nurs.

[34] Bailey SC, Andrews EN, Halton CC, Wolf MS (2021). Evaluation of a discussion guide to promote patient understanding of menopause and informed treatment decision-making. J Womens Health (Larchmt).

[35] Bailey SC, Wallia A, Wright S, Wismer GA, Infanzon AC, Curtis LM, Brokenshire SA, Chung AE, Reuland DS, Hahr AJ, Hornbuckle K, Lockwood K, Hall L, Wolf MS (2019). Electronic health record-based strategy to promote medication adherence among patients with diabetes: longitudinal observational study. J Med Internet Res.

[36] Bailey SC, Wismer GA, Parker RM, Walton SM, Wood AJJ, Wallia A, Brokenshire SA, Infanzon AC, Curtis LM, Kwasny MJ, Wolf MS (2017). Development and rationale for a multifactorial, randomized controlled trial to test strategies to promote adherence to complex drug regimens among older adults. Contemp Clin Trials.

[37] Son H, Nahm E, Zhu S, Galik E, van de Castle B, Seidl KL, Russomanno V (2022). Older adults' perception on and use of patient portals: a comparative analysis of two samples. Comput Inform Nurs.

[38] Suri S, Yoong D, Short D, Tan DH, Naccarato M, Crane HM, Musten A, Fredericksen RJ, Lober WB, Gough K (2022). Feasibility of implementing a same-day electronic screening tool for clinical assessment to measure patient-reported outcomes for eliciting actionable information on adherence to HIV medication and related factors in a busy Canadian urban HIV clinic. Int J STD AIDS.

[39] Yedulla NR, Hester JD, Patel MM, Cross AG, Peterson EL, Makhni EC (2023). Pre-visit digital messaging improves patient-reported outcome measure participation prior to the orthopaedic ambulatory visit: results from a double-blinded, prospective, randomized controlled trial. J Bone Joint Surg Am.

[40] Kroenke K, Haggstrom D, Monahan P, Kean J, Stump T, Talib T (2018). Does Sharing Patient-Reported Outcomes with Doctors Improve Patient Symptoms?.

