# Supplementary material for: Phenotyping Adherence Through Technology-Enabled Reports and Navigation (the PATTERN Study): Qualitative Study for Intervention Adaptation Using the Exploration, Preparation, Implementation, and Sustainment Framework
Source: JMIR Form Res. 2024 Oct 17;8:e54916. doi: 10.2196/54916 (PMC11528165; doi:10.2196/54916)
Supplement: Multimedia Appendix 1 [file formative_v8i1e54916_app1.pdf]

COREQ (COnsolidated criteria for REporting Qualitative research) Checklist

A checklist of items that should be included in reports of qualitative research. You must report the page number in your manuscript where you consider each of the items listed in this checklist. If you have not included this information, either revise your manuscript accordingly before submitting or note N/A.

Topic	Item No.	Guide Questions/Description	Reported on Page No.	Notes
**Domain 1: Research team and reflexivity**				
Personal characteristics				
Interviewer/facilitator	1	Which author/s conducted the interview or focus group?	N/A	Dr. Allison Pack and Ms. Evelyn Velazquez
Credentials	2	What were the researcher's credentials? E.g. PhD, MD	Title page	APP=PhD, SCB=PhD, RO=PhD, EV=BS, GW=MPH, FY=MPH, LC= MS, KA=BA, MSW=PhD
Occupation	3	What was their occupation at the time of the study?	Title Page	All researchers are affiliated with the Northwestern University Feinberg School of Medicine
Gender	4	Was the researcher male or female?	N/A	Researchers consisted of 8 females and 1 male.
Experience and training	5	What experience or training did the researcher have?	4	All were trained in research methods.
Relationship with participants				
Relationship established	6	Was a relationship established prior to study commencement?	N/A	No
Participant knowledge of the interviewer	7	What did the participants know about the researcher? e.g. personal goals, reasons for doing the research	6-7	Each participant received introductory information explaining the purpose of the interview and the institution that would be conducting the interview. Each participant read and signed a consent form explaining the study.
Interviewer characteristics	8	What characteristics were reported about the interviewer/facilitator? e.g. Bias, assumptions, reasons and interests in the research topic	N/A	Interviewers were adult women from the United States who were trained in qualitative interviewing.
**Domain 2: Study design**				
Theoretical framework				
Methodological orientation and Theory	9	What methodological orientation was stated to underpin the study? e.g. grounded theory, discourse analysis, ethnography, phenomenology, content analysis	N/A	Thematic analysis using procedures from the Rapid Identification of Themes from Audio Recording.
Participant selection				
Sampling	10	How were participants selected? e.g. purposive, convenience, consecutive, snowball	6-7	Purposive sampling was used. Recruitment occurred through partnerships with the health system.
Method of approach	11	How were participants approached? e.g. face-to-face, telephone, mail, email	6-7	Clinician participants were approached by email. Primary care patient participants were approached via mail and telephone.
Sample size	12	How many participants were in the study?	8	25

Topic	Item No.	Guide Questions/Description	Reported on Page No.	Notes
Non-participation	13	How many people refused to participate or dropped out? Reasons?	N/A	63 participants refused to participate and 1 participant withdrew from the study post-consent.
Setting				
Setting of data collection	14	Where was the data collected? e.g. home, clinic, workplace	7	Data was collected via interviews through audio-recorded secure web-conferencing software.
Presence of non-participants	15	Was anyone else present besides the participants and researchers?	N/A	No.
Description of sample	16	What are the important characteristics of the sample? e.g. demographic data, date	6-7	Demographic data included age, gender, and number of medications prescribed.
Data collection				
Interview guide	17	Were questions, prompts, guides provided by the authors? Was it pilot tested?	7	The interview guide was read by the interviewer and participants responded orally. The research team pilot tested the interview guide internally and it was informed through the Exploration, Preparation, Implementation and Sustainment (EPIS) Framework.
Repeat interviews	18	Were repeat interviews carried out? If yes, how many?	N/A	No
Audio/visual recording	19	Did the research use audio or visual recording to collect the data?	7	The interviews were audio recorded with participants' electronic consent.
Field notes	20	Were field notes made during and/or after the interview or focus group?	8	Field notes were written after each interview.
Duration	21	What was the duration of the interviews or focus group?	N/A	Each interview lasted approximately 45 minutes.
Data saturation	22	Was data saturation discussed?	8	Yes, saturation was confirmed during coding and iterative analysis
Transcripts returned	23	Were transcripts returned to participants for comment and/or	N/A	Transcripts were not returned to respondents.
**Domain 3: analysis and findings**				
Data analysis				
Number of coders	24	How many data coders coded the data?	8	Three coders coded the transcripts.
Description of coding tree	25	Did authors provide a description of the coding tree?	8	The interview guide was used to create the coding tree.
Derivation of themes	26	Were themes identified in advance or derived from the data?	8, 10-17	Themes were identified and derived from the data, but also informed by the EPIS framework.
Software	27	What software, if applicable, was used to manage the data?	8	Data was analyzed in Excel in accordance with procedures from the Rapid Identification of Themes from Audio Recordings.
Participant checking	28	Did participants provide feedback on the findings?	N/A	No
Reporting				
Quotations presented	29	Were participant quotations presented to illustrate the	10, 11, 12, 13,	Yes. For clinician participants, quotations were identified based on

Topic	Item No.	Guide Questions/Description	Reported on Page No.	Notes
		themes/findings? Was each quotation identified? e.g. participant number	14, 15, 16, 17	gender and years of experience. For Primary care patient participants, quotations were identified based on age and gender.
Data and findings consistent	30	Was there consistency between the data presented and the findings?	10-17	Yes
Clarity of major themes	31	Were major themes clearly presented in the findings?	10-17	Yes
Clarity of minor themes	32	Is there a description of diverse cases or discussion of minor themes?	10-17	Yes
