# Supplementary material for: Phenotyping Adherence Through Technology-Enabled Reports and Navigation (the PATTERN Study): Qualitative Study for Intervention Adaptation Using the Exploration, Preparation, Implementation, and Sustainment Framework
Source: JMIR Form Res. 2024 Oct 17;8:e54916. doi: 10.2196/54916 (PMC11528165; doi:10.2196/54916)
Supplement: Multimedia Appendix 2 [file formative_v8i1e54916_app2.pdf]

PATTERN

Key Informant Interview Guide: Clinician and Practice Administrators

Framework

[Exploration Planning Implementation and Sustainment (EPIS) Framework: Exploration and Early Planning Phases. This is to evaluate the needs and potential evidenced based practice fit – and to drive the decision about adoption.]

Introduction

Background:

We know that many patients face challenges taking their medication – especially when they are on multiple medications. We also know that patients don't always face the same challenges. For the PATTERN study, our goal is to help identify what type of challenge patients are facing, and then let the practice know so they can link the patient to resources that might help them overcome those challenges.

A similar study was done with patients who had received kidney transplants. Now, we're exploring how to adapt it for use in primary care – and we'd like your input on how best to do that.

Target population:

Let me first begin by telling you who the study is for. To be enrolled in our study patients will need to be aged 65 or older, have multiple chronic conditions, and polypharmacy. Specifically, we are focusing on patients who have diabetes, hypertension, and hyperlipidemia – though they may also have other conditions. Patients will also need to be prescribed 8 or more medications that they – themselves – are responsible for taking.

Intervention:

Now, I'll tell you about how the intervention works. Patients will be sent brief, monthly adherence assessments via the patient portal (MyChart). The assessments identify IF a patient has experienced a medication adherence challenge. And then, if so, the TYPE of medication adherence challenge they have experienced. Phenotyped adherence challenges are then be sent to a previously identified individual – or individuals – at the primary care practice to address as appropriate. The type of challenges may be cognitive, psychological, medical, regimen, social or economic. We'll talk more about each of these.

Any questions before we begin recording and discussing this in more detail? OK. Let's begin.

[Begin recording. State date and participant ID number.]

Outer context

Patient characteristics: Demographics of target population

1. To what extent do you think older adults with diabetes, hypertension and hyperlipidemia might benefit from a strategy to support medication adherence? Why/why not?
    - What other primary care, older adult, patient populations would benefit from this?

Innovation factors

Innovation fit: Extent to which the innovation fits the needs of the population

2. Thinking about older adult patients with diabetes, hypertension and hyperlipidemia, to what extent would you say you **already know when** they have a medication adherence challenge?
    - How would you say you know this?

3. To what extent would you say you **already know the type** of medication adherence challenge?
    - How do you know what type of challenge it is?

4. What would you say are the potential concerns – in general – related to phenotyping medication adherence challenges for this population?

5. What concerns are there for older adults using MyChart to complete regular adherence assessments to identify and phenotype their adherence challenges?

Innovation characteristics: Features of the innovation to be implemented

6. How would you address medication challenges that are **cognitive** (e.g., forgetfulness)?
    - Would you call those patients? What would you say?
    - Would you send those patients a note through the patient portal? What would it say?
    - Would you bring them into the clinic for a visit? What would you do?
    - What else might you do?
    - Which type of provider/healthcare team member is ideally suited to address this challenge?

7. How would you address medication challenges that are **psychological** (e.g., health literacy, depression, motivation)?
    - Would you call those patients? What would you say?
    - Would you send those patients a note through the patient portal? What would it say?
    - Would you bring them into the clinic for a visit? What would you do?
    - What else might you do?
    - Which type of provider/healthcare team member is ideally suited to address this challenge?

8. How would you address medication challenges that are **medical** (e.g., acute illness)?
    - Would you call those patients? What would you say?
    - Would you send those patients a note through the patient portal? What would it say?
    - Would you bring them into the clinic for a visit? What would you do?
    - What else might you do?
    - Which type of provider/healthcare team member is ideally suited to address this challenge?

9. How would you address medication challenges that are **regimen-related** (e.g., side effects, dosing schedules)?

- Would you call those patients? What would you say?
- Would you send those patients a note through the patient portal? What would it say?
- Would you bring them into the clinic for a visit? What would you do?
- What else might you do?
- Which type of provider/healthcare team member is ideally suited to address this challenge?

10. How would you address medication challenges that are **social** (e.g., transportation, support)?
    - Would you call those patients? What would you say?
    - Would you send those patients a note through the patient portal? What would it say?
    - Would you bring them into the clinic for a visit? What would you do?
    - What else might you do?
    - Which type of provider/healthcare team member is ideally suited to address this challenge?

11. How would you address medication challenges that are **economic** (e.g., costs)?
    - Would you call those patients? What would you say?
    - Would you send those patients a note through the patient portal? What would it say?
    - Would you bring them into the clinic for a visit? What would you do?
    - What else might you do?
    - Which type of provider/healthcare team member is ideally suited to address this challenge?

Inner context

Organizational characteristics: Readiness for change

12. How ready would you say your practice is for this type of intervention – one that uses the patient portal to routinely phenotype medication adherence concerns and inform the practice?
    - What changes does your practice need to make to be ready?

Individual characteristics: Job demands

13. Given competing job demands, how difficult will it be for existing care team member(s) to prioritize addressing the medication adherence challenges they are informed of through PATTERN?
    - What would help them to address those challenges?

Conclusion

14. Thank you. What else should we be thinking about that we haven't discussed related to adapting our intervention for use in primary care?

Key Informant Interview Guide: Patients

Framework

[Exploration Planning Implementation and Sustainment (EPIS) Framework: Exploration and Early Planning Phases. This is to evaluate the needs and potential evidenced based practice fit – and to drive the decision about adoption.]

Introduction

Background:

We know that many patients face challenges taking their medication – especially when they are on multiple medications. We also know that patients don't always face the same challenges. For example, for some patients, their biggest challenge may be remembering to take their medications, while for others, their biggest challenge may be the medication cost. For the PATTERN study, our goal is to help identify what type of challenge patients are facing, and then let the health care practice know so they can link the patient to resources that might help them overcome those challenges.

A similar study was done with patients who had received kidney transplants. Now, we're exploring how to adapt it for use in primary care – and we'd like your input on how best to do that.

Target population:

Let me first begin by telling you who the study is for. To be enrolled in our study patients, will need to be aged 65 or older. Patients will also need to have diabetes, hypertension, and hyperlipidemia – though they may also have other conditions. And finally, patients will need to be prescribed 8 or more medications that they – themselves – are responsible for taking. These are medications that they are **already** taking since they will not be given any medication as part of the study.

Intervention:

Now, I'll tell you about how the intervention works. Patients are sent monthly medication adherence assessments (or questionnaires) through the patient portal. The assessments take around 5 minutes to complete. They first identify IF, in the past month, the patient has experienced any challenges taking their medications the way their doctor asked them to. (We refer to those challenges as medication adherence challenges.) If a patient *has* experienced challenges, the assessment will help identify the TYPE of challenge they have experienced. The type of challenge may be cognitive (forgetfulness), psychological (that's things like motivation), medical (if they're suffering from acute illness), regimen (that has to do with side effects and when patients must take their medications), social (needing support) or economic (that's cost). We'll talk more about each of these in more detail. But it's important to know that the patient's health care team will be told what type of challenge the patient has experienced. This way, they can help address it.

Any questions before we begin recording and discussing this in more detail? OK. Let's begin.

[Begin recording. State date and participant ID number.]

Outer context

Patient characteristics: Demographics of target population

1. To what extent do you think older adults living with multiple chronic health conditions – like diabetes, hypertension and hyperlipidemia – might benefit from a strategy that helps them better manage their medication adherence? Why/why not?
    - To what extent do **you** think you would benefit from such a strategy?

Innovation factors

Innovation fit: Extent to which the innovation fits the needs of the population

2. To what extent would you say your healthcare team **already knows when** you have a medication adherence challenge? This would be any challenge you have taking your medication the way your doctor asked you to.
    - Why is that?

3. To what extent would you say your healthcare team **already knows the type** of medication adherence challenge(s) you have?
    - Why is that?

4. How would you feel about having to complete brief, monthly adherence assessments (short questionnaires)?
    - What would make you more likely to complete them?
    - How do you think others would feel?

5. What concerns, if any, do you have about using the patient portal (MyChart) to complete those assessments? To do this, you will receive an email saying you have a questionnaire to complete in MyChart. You'd then need to login to MyChart and answer the questions. You would receive a reminder email if you forgot to complete the assessment within a week.

Innovation characteristics: Features of the innovation to be implemented

6. How would you want your health care team to help you address medication challenges that are **cognitive** (e.g., forgetfulness)?
    - Would you want them to call you? What would you want them to say?
    - Would you want to receive a note through the patient portal? What would you want it to say?
    - Would you want to meet with a health care provider? What would you want them to do?

7. How would you address medication challenges that are **psychological** (e.g., health literacy, depression, motivation)?
    - Would you want them to call you? What would you want them to say?
    - Would you want to receive a note through the patient portal? What would you want it to say?

- Would you want to meet with a health care provider? What would you want them to do?

8. How would you address medication challenges that are **medical** (e.g., acute illness)?
   - Would you want them to call you? What would you want them to say?
   - Would you want to receive a note through the patient portal? What would you want it to say?
   - Would you want to meet with a health care provider? What would you want them to do?

9. How would you address medication challenges that are **regimen-related** (e.g., side effects, dosing schedules)?
   - Would you want them to call you? What would you want them to say?
   - Would you want to receive a note through the patient portal? What would you want it to say?
   - Would you want to meet with a health care provider? What would you want them to do?

10. How would you address medication challenges that are **social** (e.g., transportation, support)?
    - Would you want them to call you? What would you want them to say?
    - Would you want to receive a note through the patient portal? What would you want it to say?
    - Would you want to meet with a health care provider? What would you want them to do?

11. How would you address medication challenges that are **economic** (e.g., costs)?
    - Would you want them to call you? What would you want them to say?
    - Would you want to receive a note through the patient portal? What would you want it to say?
    - Would you want to meet with a health care provider? What would you want them to do?

Conclusion

12. What else should we be thinking about that we haven't discussed related to adapting our adherence intervention for use in primary care?
